# New screw technique for posterior column and ischial fixation from the anterior approach for acetabular fractures

**DOI:** 10.1093/jscr/rjad073

**Published:** 2023-02-27

**Authors:** Kunihiro Asanuma, Gaku Miyamura, Yoshiaki Suzuki, Yoshinori Makino, Naoya Takada, Haruhiko Satonaka, Kakunoshin Yoshida, Tomohito Hagi, Tomoki Nakamura, Akihiro Sudo

**Affiliations:** Department of Orthopedic Surgery, Mie University School of Medicine, Tsu, Japan; Department of Orthopedic Surgery, Mie University School of Medicine, Tsu, Japan; Department of Orthopedic Surgery, Mie University School of Medicine, Tsu, Japan; Department of Orthopedic Surgery, Mie University School of Medicine, Tsu, Japan; Department of Orthopedic Surgery, Kainan Hospital, Yatomi, Japan; Department of Orthopedic Surgery, Ise Municipal General Hospital, Ise, Japan; Department of Orthopedic Surgery, Ise Municipal General Hospital, Ise, Japan; Department of Orthopedic Surgery, Mie University School of Medicine, Tsu, Japan; Department of Orthopedic Surgery, Mie University School of Medicine, Tsu, Japan; Department of Orthopedic Surgery, Mie University School of Medicine, Tsu, Japan

**Keywords:** acetabular fractures, posterior column, ischium, quadrilateral plate, sleeve guide technique

## Abstract

Management of the ischial fragment in acetabular fractures is a considerable problem. In this report, we presented how to drill or screw around the posterior column and ischium from the anterior approach using a novel ‘sleeve guide technique’ and the difficulty of plating. A sleeve, drill, depth gauge and driver from DepuySynthes were prepared. The portal was about 2–3 cm inside the anterior superior iliac spine opposite to the side of the fracture. The sleeve was inserted to the screw point around quadrilateral area through the retroperitoneal space. Drilling, measuring screw length by a depth gauge and the screwing were performed through the sleeve. Case 1 used a one-third plate and case 2 used a reconstruction plate. With this technique, the approach angles to the posterior column and ischium were inclined, and plating and screw insertion could be performed with a low risk of organ injury.

## INTRODUCTION

Acetabular fractures are relatively common, but their surgical treatment is still challenging. Anterior column and posterior hemitransverse fractures, T-type fractures and both column fractures are particularly difficult and complicated to reduce and fix. Depending on the fracture complexity, anterior fixation, posterior fixation or both have been performed [1]. From the anterior approach, the management of the ischial or posterior column fragment is a considerable problem, and it is important to determine whether to use only the anterior approach or to add the posterior approach.

To fix the ischial or posterior fragment from the anterior approach, a couple of techniques are known, as discussed below. Posterior column screws or quadrilateral area screws have usually been chosen [2]. If the fracture is associated with medial displacement of the quadrilateral surface, the major technique is buttress plating [3, 4], and a unique technique is buttress screws on the inner surface of the quadrilateral plate [5] or wire plate composite fixation [6].

**Figure 1:**
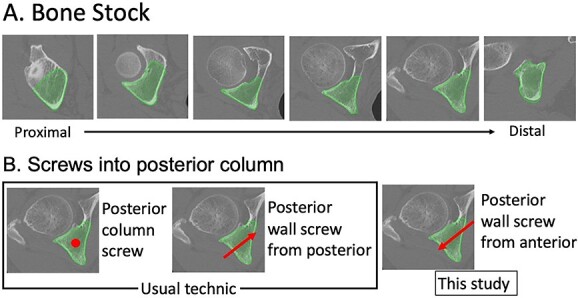
Bone stock and screw into the posterior column. Thick and strong bone stock in the posterior column is shown from proximal to distal. The screw direction is shown by a red dot or arrows. The posterior column screw is indicated by a dot because the screw is inserted into the posterior column from proximal to distal.

In the posterior column, thick and strong bone stock exists from proximal to distal (Fig. 1).

The screw zone is wide, and the usual technique to screw into the posterior column is a posterior column screw or a posterior wall screw from the posterior. However, a screw into the posterior column, especially the distal area, from the anterior has not been reported (Fig. 1). If the ischial or posterior column fragment can be fixed solidly from the anterior approach, it may reduce the need for posterior incision. If a quadrilateral fragment can be fixed by inserting screws into the posterior column and ischium, re-dislocation may be reduced, and the timing of loading may be accelerated.

**Figure 2:**
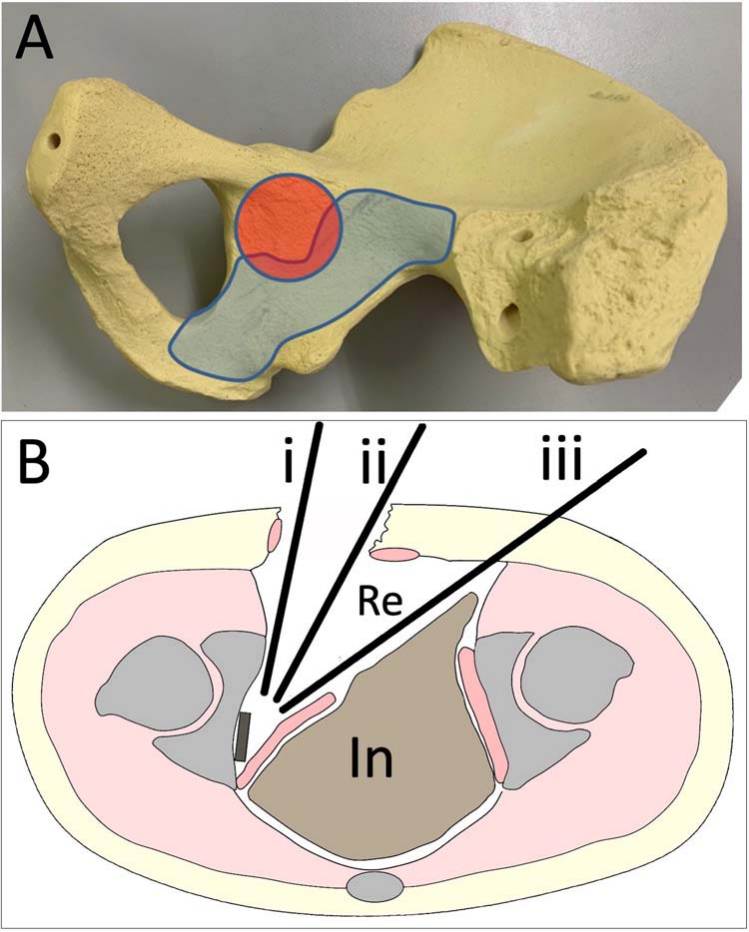
Approach images (**A**). The red area indicates the acetabular joint. In the blue area, screws can be inserted (**B**). (i) Drilling and screwing direction of the ilioinguinal or pararectus approach. (ii) Drilling and screwing direction of the modified Stoppa approach. (iii) Drilling and screwing direction with the sleeve guide technique. Re, retroperitoneal area. In, intra-abdominal area.

Detaching the internal obturator muscle from the quadrilateral plate is a common technique to set a buttress plate. However, inserting a screw to the plate near the posterior column and ischium has not been reported, despite the thick and strong bone mass there. Recently, He et al. reported a study of the safe zone around this area [7]. We considered the safe zone for a screw as the blue area in Fig. 2(A), and the difficulty in inserting a screw to the plate arises from not only the approach angle to the plate by drilling or screwing, but also the risk of pelvic organ injury (Fig. 2B). Although the drilling or screwing approach angle is dependent on the surgical approach and the skin incision, the angle is too sharp to insert into a plate hole (Fig. 2Bi, ii). The current report is the first to demonstrate a novel ‘sleeve guide technique’ involving tilting the approach angle of the screw or drill around the posterior column and ischium by making a safe route with a sleeve through a portal on the opposite side of the abdomen (Fig. 2Biii).

## SCREW DIRECTION

**Figure 3:**
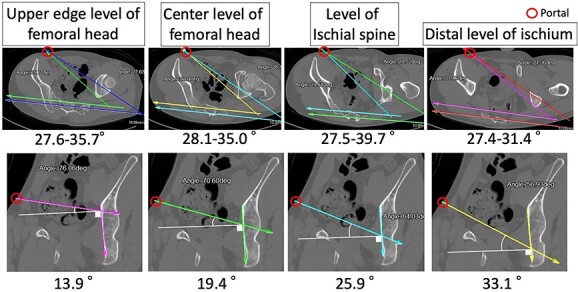
Estimated screw angles, Example CT images are shown from the upper edge level of the femoral head to the distal part of the ischium. The upper row shows inclined axial plane images, and the lower row shows inclined coronal plane images. Estimated screw angles to plate from the portal site to the posterior column are measured.

**Figure 4:**
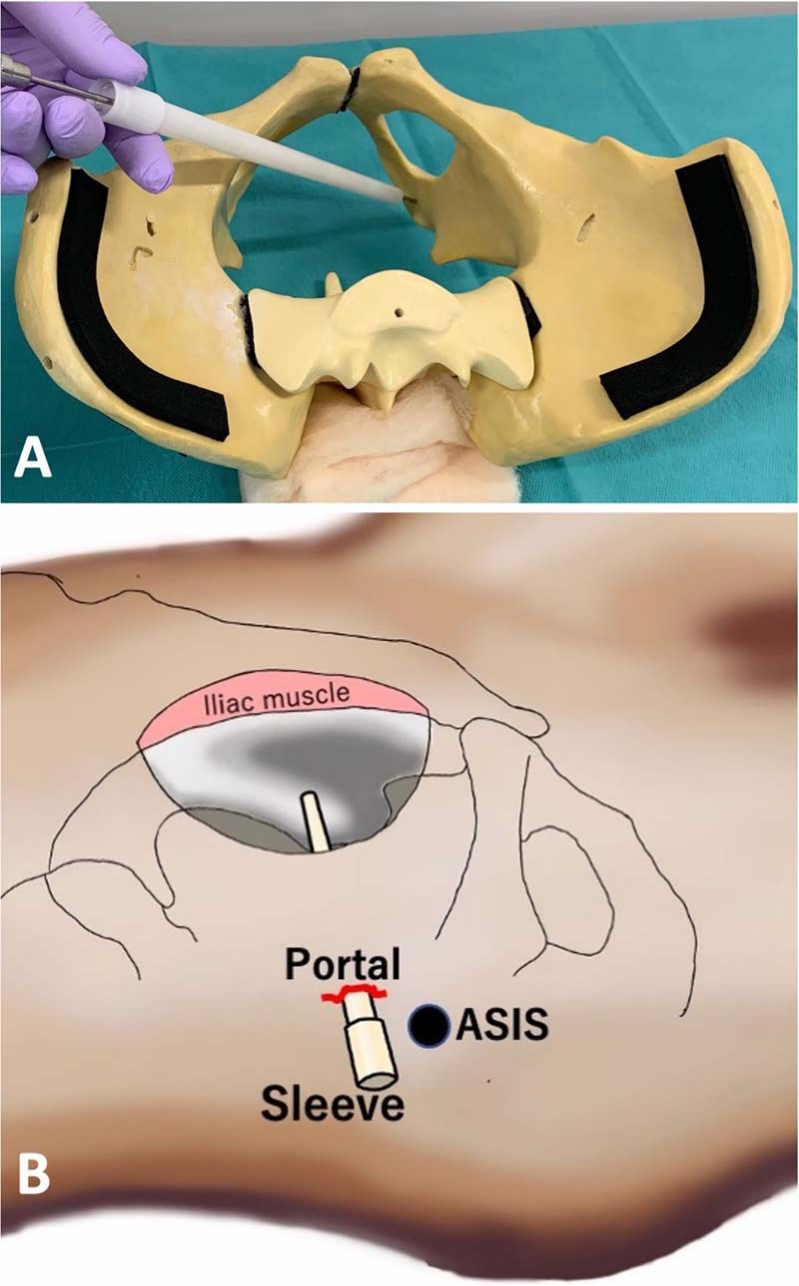
Actual direction of the sleeve using a pelvic model (**A**) and scheme (**B**).

This technique adjusts the approach angle of the screw or drill to the plate around the posterior column and ischium by making a portal around the anterior superior iliac spine (ASIS) on the opposite side of the abdomen (Fig. 2Biii). As an example, the approach angle range of the screw from the insertion point around the ASIS to the posterior column was measured by computed tomography (CT). To measure the approach angle, 3D-CT viewer (AquariusNET viewer, V4.4.13, P2, TeraRecon, Inc. Durham, NC, USA) was used to show the route from the portal site to the posterior column in the same image in an inclined axial plane (Fig. 3). At the upper edge level of the femoral head, the angle range was 27.6°–35.7°. At the center level of the femoral head, the angle range was 28.1°–35.0°. At the level of the ischial spine, the angle range was 27.5°–39.7°. At the distal level of the ischium, the angle range was 27.4°–31.4°. In the inclined coronal plane, the insertion point and posterior wall are shown. The angle to the upper edge level of the femoral head was 13.9°. The angle to the center level of the femoral head was 19.4°. The angle to the level of the ischial spine was 25.9°. The angle to the distal level of the ischium was 33.1°. Sleeve direction using a model is shown in Fig. 4(A, B). These data are presented to understand the drilling and screw angles as an example.

## SLEEVE GUIDE TECHNIQUE

**Figure 5:**
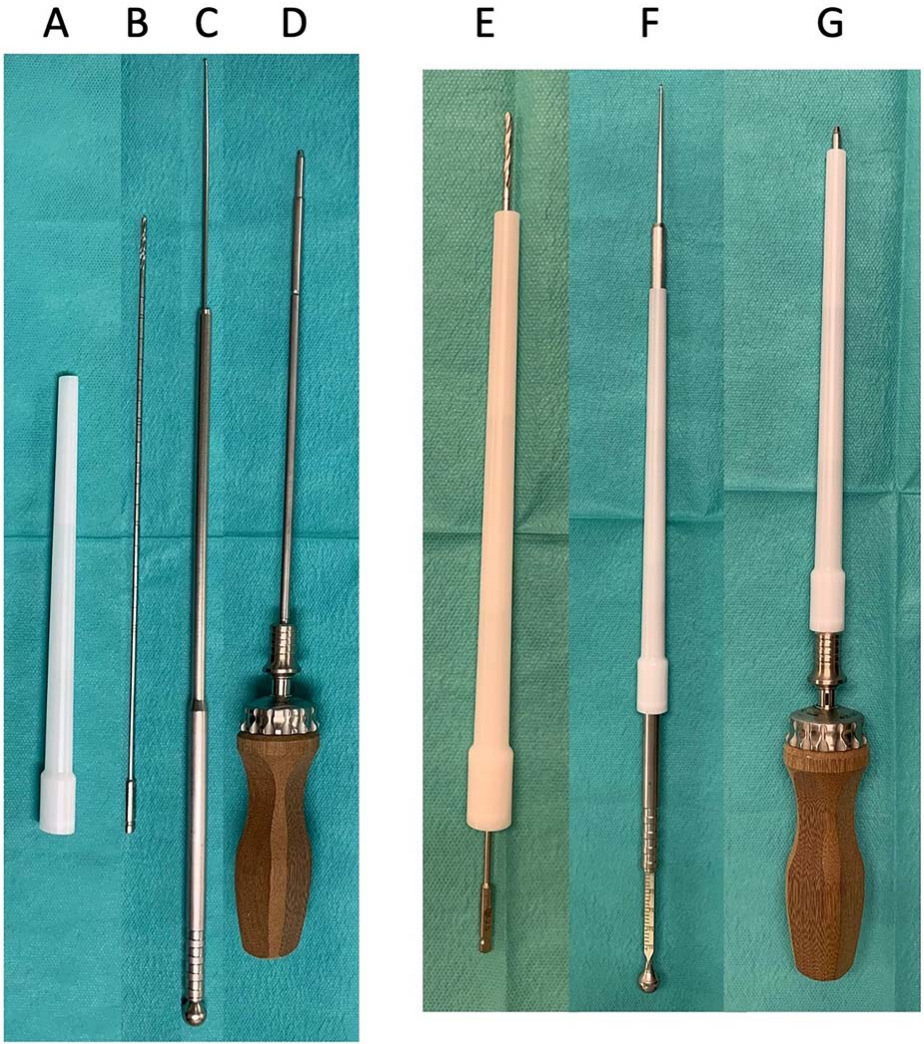
Devices for the sleeve guide technique: (**A**) sleeve, (**B**) drill, (**C**) depth gauge, (**D**) driver. Product name and number are in the text. (**E**) Insert drill into the sleeve, (**F**) insert depth gauge into the sleeve, (**G**) insert the driver into the sleeve.

A sleeve (Fig. 5A, No.831230, DepuySynthes, CEMVAC 8.5 REVIS NOZZLE), Drill (Fig. 5B, No. 324-210, Drill Bit φ2.5 mm, calibrated, length 300 mm, with Quick Coupling, for Percutaneous Insertion), depth gauge (Fig. 5C, No. 356-835, Measuring Device for Locking Bolt) and driver (Fig. 5D, No. 03-100-032, Ratcheting Handle with AO/ASIF Quick Coupling, No. 03-100-033, Screwdriver Shaft, hexagonal, for Screws φ3.5 mm, length 250 mm) were prepared. All products were from DepuySynthes.

Briefly, after detaching the internal obturator muscle, the periosteum was elevated from the quadrilateral plate. The obturator muscle was retracted, paying attention to avoid injuring the obturator nerve and vascular structures. The plate was bent and adapted to the pelvic surface. First, the skin was cut about 2–3 cm inside of the ASIS opposite the side of the fracture, and a hand was slipped between the abdominal muscle and peritoneal membrane from the surgical field just below the skin incision. The subcutaneous fat and all abdominal muscles were divided toward the hand using a mosquito clamp or other tool. The nozzle was inserted as an external sleeve and grabbed, and the sleeve head was led to the screw point. The sleeve head was adjusted to aim at the screw insertion point. Video 1 shows the obturator nerve over the sleeve. After drilling, the hip joint movement was confirmed to check femoral head insertion before pulling it out (Fig. 5E, Video 2). A C-arm oblique image in the drill direction can be used to determine whether the drill is inside or outside the acetabular joint. Intraoperative CT in the drilling direction is shown in Video 3. Screw length was measured by a depth gauge for proximal femoral nail anti-rotation (PFNA) (Fig. 5F, Video 4), and the screw and driver were inserted (Fig. 5G, Video 4) through the sleeve. The screw could be held near the drilling hole and set to the driver and screwed in. With this technique, one can drill and insert the screw to the blue area through the retroperitoneal space with a low risk of organ injury (Fig. 2A).

## CASE SERIES

### Case 1

**Figure 6:**
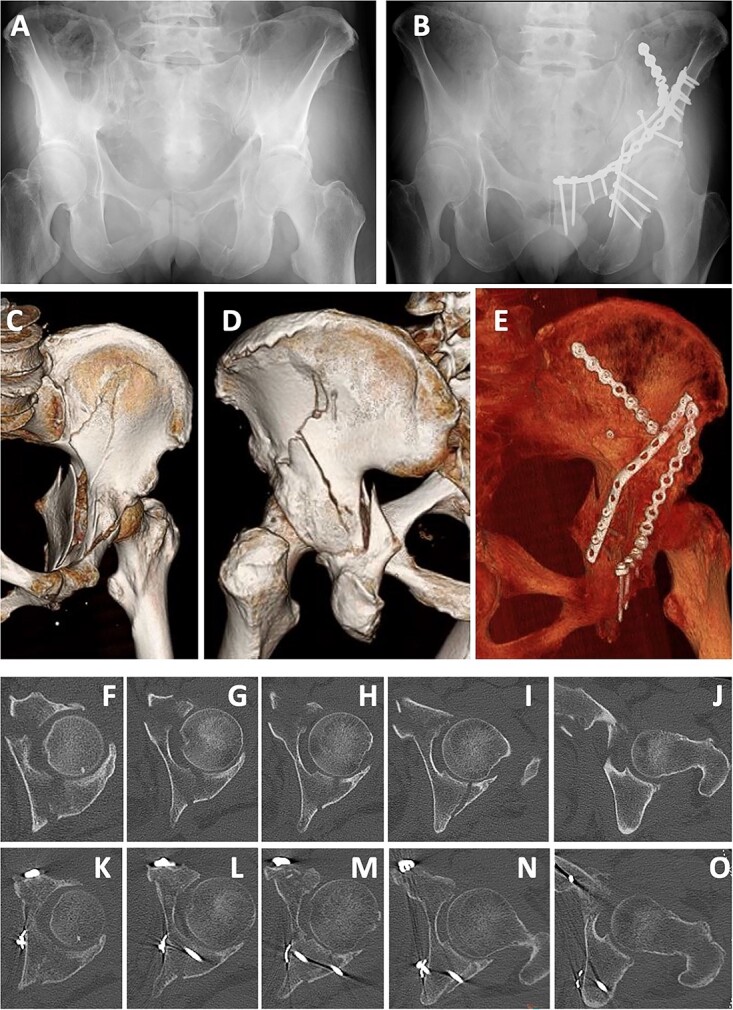
Case 1 images: (**A**) pre-operative X-ray, (**B**) post-operative X-ray, (**C**) pre-operative 3D-CT (anterior), (**D**) pre-operative 3D-CT (posterior), (**E**) post-operative 3D-CT, (**F**–**J**) pre-operative axial CT images, (**K**–**O**) post-operative axial CT.

A 55-year-old man was involved in a motorcycle accident and taken to a nearby hospital. Diagnostic imaging showed a left acetabular fracture (Fig. 6A, C, D, F–J; both column fractures according to the AO classification). He was transferred to our hospital, and 12 days after the injury, open reduction and internal fixation were performed using a low-profile pelvic plate system (DePuySynthes Trauma, West Chester, PA, USA). The patient was placed in the supine position, and the classical ilioinguinal approach was used. The skin was cut from the pubic joint to the iliac crest, the abdominal muscles were released, and the retroperitoneal area and the fracture area were reached. Dislocation of the pelvic brim was reduced with a reduction clamp and fixed by a screw. After the internal obturator muscle was retracted, the quadrilateral fragment was reduced, a bent 12-hole, one-third plate was placed at the quadrilateral surface, and a screw was inserted from the posterior column to the ilium using the sleeve guide technique (Fig. 6K–O). In addition, two reconstruction plates were used for fixation from the anterior to the posterior ilium and from the pubis to the ilium (Fig. 6B, E). Six weeks after the operation, partial weight-bearing was started. After 3 months, the patient walked with a stick. After 1 year, he reported no hip or pelvic pain, and he walked without a gait abnormality.

### Case 2

**Figure 7:**
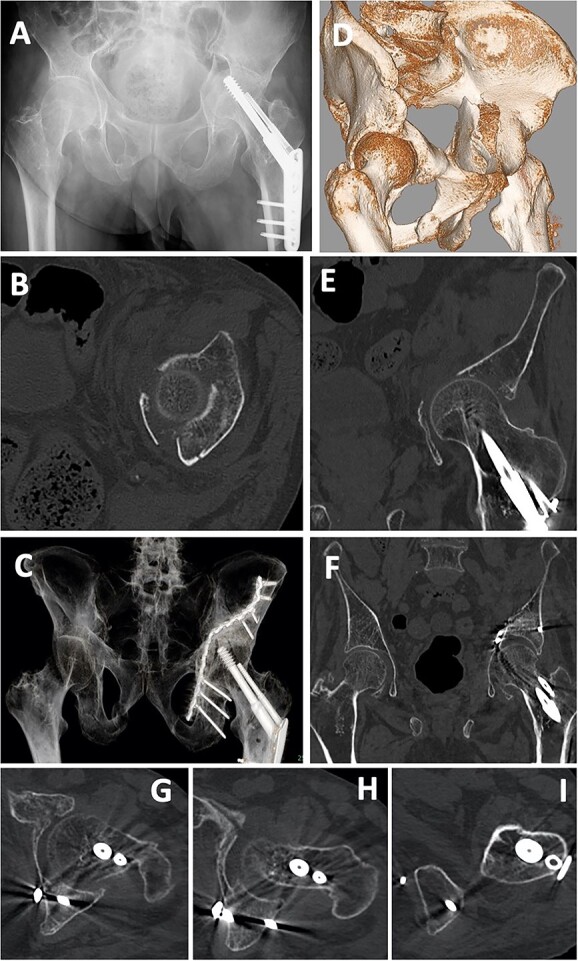
Case 2 images: (**A**) pre-operative X-ray, (**B**) pre-operative axial CT image, (**C**) post-operative X-ray, (**D**) pre-operative 3D-CT, (**E**) pre-operative coronal CT image, (**F**) post-operative 3D-CT, (**G**–**I**) post-operative axial CT images. Screws are inserted into the posterior wall (G, H) and near the ischial tuberosity (I).

A 66-year-old man, who walked with a stick, was injured in a fall during a sudden convulsion. He could not move his left lower extremity due to pain and rested in his house. After 3 days, he developed a fever (>38°C) and was admitted to a nearby hospital. Diagnostic imaging showed a quadrilateral fracture (Fig. 7A, B, D, E). Twelve days after injury, open reduction and internal fixation for the acetabular fracture were performed using a low-profile pelvic plate system by the classical ilioinguinal approach, as in case 1. After a 13-hole reconstruction plate was bent and placed at the quadrilateral surface, screws were inserted to the posterior column and ischium using the sleeve guide technique (Fig. 7C, F–I). Six weeks after the operation, partial weight-bearing was started. After 3 months, he was able to walk with a rollator, and after 1 year, he walked with a stick.

## DISCUSSION

With this technique, the approach angle of the screw or drill to the plate on the posterior column and ischium is inclined (Fig. 2B), which makes it possible to fix a plate to the posterior column or ischium by making the insertion point on the opposite side of the abdomen. Furthermore, a dislocated quadrilateral plate can be fixed or compressed rigidly by fixation at both ends using this technique. The operative indications for the fracture types have not been clear. In our opinion, this technique can be used for fractures that require not only anterior fixation, but also posterior fixation, or a dislocated quadrilateral fracture. A posterior wall fracture with wide dislocation needing reduction is not suitable. A study of the safe zone of the quadrilateral surface suggested the possibility of a two-ended plate as a viable alternative to single-ended elastic fixation for quadrilateral fractures [7]. The present technique makes it possible to fix a quadrilateral fracture with a two-ended plate.

This technique requires understanding of the retroperitoneal area, as the sleeve is handled through this area. Any approach that opens the retroperitoneal area and the quadrilateral space, such as the ilioinguinal approach [8], modified Stoppa approach [9, 10], pararectus approach [11], anterior intrapelvic approach or supra-ilioinguinal approach [12], is thought to be possible for this technique. In general, the approach angle to the plate by the drill or driver is closer to vertical in the modified Stoppa approach (Fig. 2Bii) than in other approaches (Fig. 2Bi). However, the angle to the plate with the present technique is closer to vertical (Fig. 2Biii) than any other approach, including the modified Stoppa approach.

**Figure 8:**
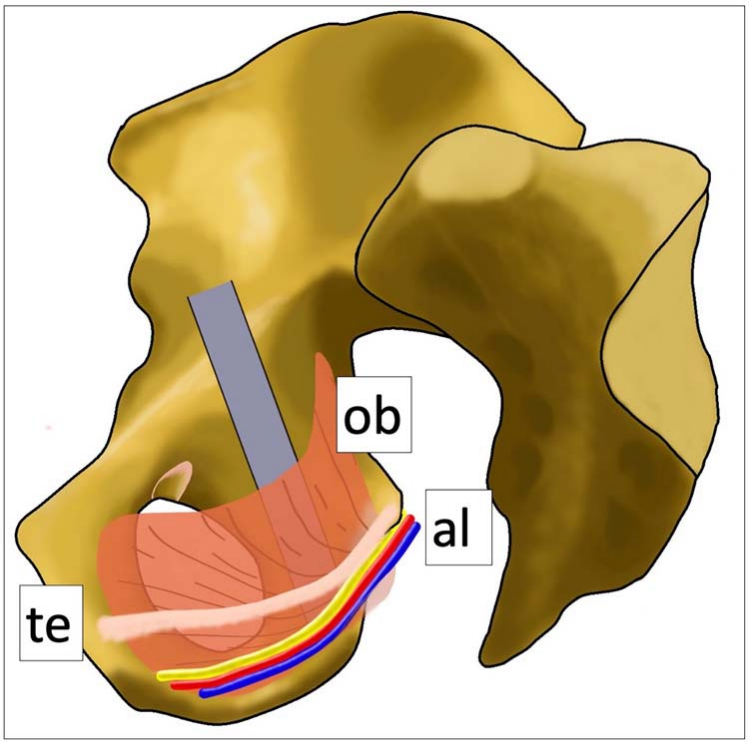
Anatomical schematic diagram. This schematic diagram shows the anatomical relationships of the internal obturator muscle, Alcock’s canal and the tendinous arch of the levator ani muscle. Ob: internal obturator muscle, al: Alcock’s canal (pudendal vessels and nerve), te: tendinous arch of the levator ani muscle.

In this technique, there are some tricks and traps. First, a sharp retractor should not be inserted into the obturator muscle near the ischial tuberosity and the lower end of the quadrilateral space. Alcock’s canal, including the pudendal vessels and nerve, is folded inside the wall of the obturator muscle [13, 14], and attention must be paid not to injure them (Fig. 8). The pudendal nerve, which has three branches (inferior rectal nerve, perineal nerve, dorsal nerve of the penis in males, and dorsal nerve of the clitoris in females), is associated with urinary and sexual function [15]. Second, rough retraction of obturator nerve should be avoided. Third, as the sciatic nerve is close to the posterior wall, overdrilling must be avoided.

## CONCLUSION

A novel ‘sleeve guide technique’ to insert screws to the posterior column and ischium from the anterior approach by inclining the approach angle of drilling and screwing was presented. The methods are simple, safe and useful, and the four devices required are all easily available from DePuySynthes and used worldwide for fracture surgery. However, reaching the posterior column and ischium from the anterior approach requires detailed anatomical knowledge and surgical experience. Our technique provides the methodology on how to insert the screws around the posterior column and ischium from the anterior approach. To receive a benefit from this technique, a straight plate is not enough. Pro pelvis and Acetabular System (Striker) is one choice. New plate development to cover the quadrilateral plate and posterior column and ilium is needed.

## LIMITATION

As this was a preliminary study that showed the utility of the sleeve guide technique and how to drill and screw to the posterior column and ischium from the anterior approach with two case reports, complications and functional results need to be investigated in a case series or a case-control study.

## CONFLICT OF INTEREST STATEMENT

None declared.

## FUNDING

There was no funding toward this research.

## DATA AVAILABILITY

The data that support the findings of this study are available from the corresponding author upon reasonable request.

## AUTHORS' CONTRIBUTIONS

K.A. operated on all patients and wrote the main manuscript text and prepared all figures and tables. G.M., Y.M., T.N. and T.H. joined the operation of case 1 and contributed to data collection. Y.S., H.S. and K.Y. joined the operation of case 2 and contributed to data collection. N.T. critically reviewed the manuscript. A.S. supervised this study. All authors have read and approved the manuscript. All authors issued the final approval for the version to be submitted.

## Supplementary Material

video_1_rjad073

video_2_rjad073

video_3_rjad073

video_4_rjad073
